# Impact of air injection on subretinal fluid following successful scleral buckling surgery for macular-involving retinal detachment

**DOI:** 10.1038/s41598-021-88670-1

**Published:** 2021-04-27

**Authors:** Fen Tang, Fan Xu, Ning Su, Lingjuan Liu, Li Jiang, Ningning Tang, Xin Zhao, Ling Cui, Siming Zeng, Zhaoguang Lai, Min Li, Haibin Zhong

**Affiliations:** Department of Ophthalmology, The People’s Hospital of Guangxi Zhuang Autonomous Region, Nanning, Guangxi China

**Keywords:** Medical research, Epidemiology, Outcomes research

## Abstract

Air injection is an accessory technique during scleral buckling (SB). Subclinical subretinal fluid (SRF) may presence and persistent after SB. The impact of air injection on SRF is unclear. In the study, we retrospectively enrolled 51 patients with macular-involving RD who had undergone successful SB. They were categorized into Group A (SB without air injection) and Group B (SB with air injection). First, we found that although group B seem to be severer than group A before surgery, Kaplan–Meier graph showed that SRF absorbed more rapidly in group B after surgery, and the incidence of SRF in group B was much lower during the whole follow-up period. Moreover, the cases with superior breaks had the lowest incidence. Second, during the follow-up period, there was no significant difference about postoperative complication between two groups. Lastly, risk factors for persistent SRF were investigated with binary logistic regression, and no risk factor was found. In conclusion, air injection during the SB might accelerate SRF absorption and reduce the incidence of persistent SRF, especially for the longstanding macular-off RD with superior breaks.

## Introduction

Scleral buckling (SB) is a surgical approach to repair rhegmatogenous retinal detachment (RD)^1^. However, subretinal fluid (SRF) at fovea recognized by optical coherence tomography (OCT) may present and persist for a long time after surgery, even the retina seems to be reattached on ophthalmoscopy^2^. Previous studies reported that the incidence of SRF following successful retinal detachment surgery varied ranged from 0 to 94%. It was demonstrated that persistent SRF at fovea might delay central vision recovery, and even cause irreversible visual loss^3^. However, the possible etiology and the risk factors of foveal SRF are still uncertain.

It had been identified that several factors were associated with the occurrence of SRF, such as the type of surgery and the surgical technique^3^. Although pars plana vitrectomy (PPV) is developing rapidly and gaining popularity for RD patients, SB still has several advantages over PPV, such as prevention of cataract progression and early visual rehabilitation^1,4^. However, SB is not a one-size-fits-all technique, it requires careful buckle selection to ensure success. In addition, the surgeon need to select accessory techniques appropriately, such as intravitreal gas injection, intravitreal balanced salt solution, gas-fluid exchange and laser therapy^5^. Previous studies reported that the fovea is compressed and stretched with the gas bubble inside the eyes and postoperative posturing^6,7^. However, there was no clear-cut evidence that the bubble could benefit SRF absorption. The present study was aimed to compare the foveal SRF incidence in macular-off RD after SB without intravitreal air injection or with intravitreal air injection, and to investigate the effect of air bubble on foveal SRF absorption. Besides, we also aimed to evaluate the potential risk factors for persistent SRF at fovea.

## Results

### Demographic characteristics and clinical features

**Figure 1:**
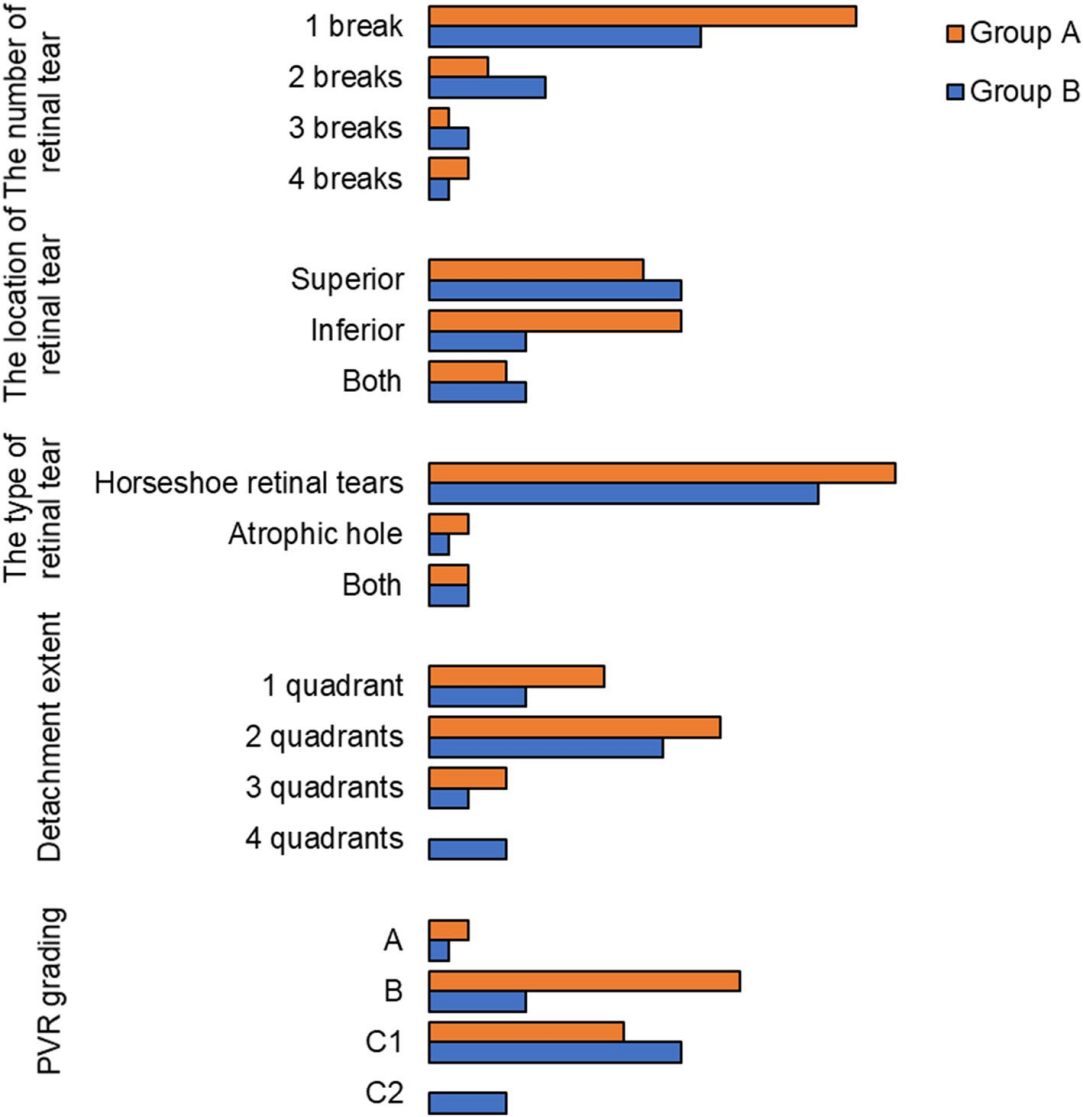
The distribution of retinal detachment features. The number of retinal breaks was evaluated and determined by three consultants. The superior break was defined as a tear present between 9 to 3 o’clock, while an inferior break was defined as a tear present between 3 to 9 o’clock. The extent was determined with the clocks of detachment, the extent less than 3 clocks was defined as 1 quadrant, the extent between 3 to 6 clocks was defined as 2 quadrants, the extent between 6 to 9 clocks was defined as 3 quadrants, and the extent between 9 to 12 clocks was defined as 4 quadrants. The grade of PVR was determined according to the classification from the American Retina Terminology Committee (1983).

Patient demographics and preoperative clinical characteristics are summarized in Table 1. The distribution of preoperative clinical characteristics was presented in Fig. 1. There were no significant differences in age, gender distribution, clinical features of retinal tears, detachment extent, or co-existing diseases. The average symptom duration of group B appeared to be longer than that of group A; however, no statistically significant difference was shown (*p* = 0.252).There were statistically significant difference between the two groups in terms of preoperative BCVA, IOP and PVR grading, which indicated that the group B had worse BCVA, lower IOP and severer PVR at baseline, compared with group A. In addition, all the cases had no preoperative choroidal detachment.

**Table 1:** Patients’ demographic and preoperative clinical characteristics.

	Group A (n = 28)	Group B (n = 23)	*p* Value
**Age, years (Mean ± SD)**	37.82 ± 14.07	43.96 ± 17.07	0.166^a^
**Gender, Female (%)**	9 (32.14%)	9(39.13%)	0.751^b^
**Symptom duration (days)**	17.89 ± 10.97	30.74 ± 31.22	0.252^c^
**BCVA (Log MAR)**	0.82 ± 0.35	1.14 ± 0.23	0.003^a^
**IOP (mmHg)**	14.11 ± 3.26	12.23 ± 2.38	0.025^a^
**Retinal breaks**			0.425^d^
1 break	22 (78.6%)	14 (60.9%)	
2 breaks	3 (10.7)	6 (26.1%)	
3 breaks	1 (3.6%)	2 (8.7%)	
4 breaks	2 (7.1%)	1 (4.3%)	
**The type of retinal tears**			1.000^d^
Tractional tear	24 (85.7%)	20 (87.0%)	
Atrophic hole	2 (7.15%)	1 (4.3%)	
Both	2 (7.15%)	2 (8.7%)	
**Location of breaks**			0.177^d^
Superior	11 (39.3%)	13 (56.5%)	
Inferior	13 (46.4%)	5 (21.7%)	
Both	4 (14.3%)	5 (21.7%)	
**Extent of detachment**			0.152^d^
1 quadrant	9 (32.1%)	5 (21.7%)	
2 quadrants	15 (53.6%)	12 (52.2)	
3 quadrants	4 (14.3%)	2 (8.7%)	
4 quadrants	0 (0%)	4 (17.4%)	
**The grade of PVR**			0.009^d^
A	2 (7.1%)	1 (4.3%)	
B	16 (57.1%)	5 (21.7%)	
C1	10 (35.7%)	13 (56.6%)	
C2	0 (0%)	4 (17.4%)	
**Co-existing diseases**			
Diabetes	1	2	0.583^d^
Hypertension	2	2	1.0^d^

^a^*t*-test.

^b^Chi-square test.

^c^Mann-Whitney U test.

^d^Fisher exact test,

Abbreviation: BCVA, Best Corrected Visual Acuity; IOP, Intraocular Pressure; PVR, Proliferative vitreoretinopathy.

Moreover, the information about buckle configuration had been extracted from surgery record. As was shown in Table 2, all the subjects were treated with encircling to relieve the forces of vitreous traction. And a segmental buckle (silicone tire #276, with a 8 mm width of mattress suture) was placed on the sclera appropriately to support closure of the break. There were no significant differences in the length of silicone tire between group A and group B. In addition, viscosity of SRF was also recorded, and we found that viscosity of the drained fluid was closely related with detachment duration, the SRF of a long-duration RD is more viscous. The percentage of viscous SRF in group B was higher than that in group A. However, there was no statistically difference (*p* = 0.154).

**Table 2:** The information of buckle configuration during surgery.

	Group A (n = 28)	Group B (n = 23)	*p* Value
**Encircling (%)**	100%	100%	
**The length of silicone tire (mm)**	16.71 ± 3.63	17.35 ± 3.47	0.530^a^
**Viscosity of SRF**			0.154^b^
Viscous	3 (10.71%)	7 (30.43%)	
Clear	25(89.29%)	16(69.57%)	

^a^*t*-test.

^b^Fisher exact test.

### The effect of air injection on SRF absorption

**Figure 2:**
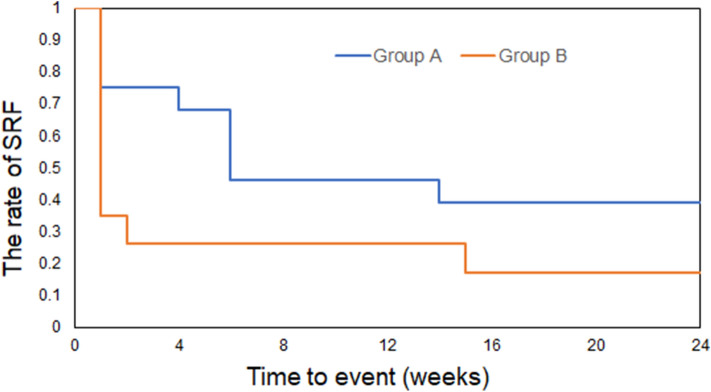
Kaplan–Meier survival curve illustrating the rate of SRF against time for group A (SB without air injection) and group B (SB with air injection). The incidence of SRF was significantly different (*p* < 0.001, log-rank test).

Then, we evaluated the incidence of SRF during the postoperative follow-up period in both groups. The presence of SRF was defined by OCT. The Kaplan–Meier survival graph was plotted to illustrate the incidence of SRF against time. As was presented in Fig. 2, at postoperative 1 week, SRF in 25% cases of group A and 65% cases of group B resolved following SB surgery, and the rate of SRF in group A was significantly higher than that in group B during the postoperative follow-up period in total (*p* = 0.000, Log rank mantel-Cox). There still had 39% cases of group A and 17% cases of group B who had persistent SRF at postoperative 24 weeks.

In addition, the incidence of persistent SRF at postoperative 24 weeks was also compared in patients with different tear location. As was shown in Table 3, for both group A and group B, the patients with both superior and inferior break had the highest incidence, and the patients with superior break had the lowest incidence. However, statistical analysis showed that there was no significant difference in group A (*p* = 0.292), while the difference was statistical significant in group B (*p* = 0.012). Moreover, the incidence of persistent SRF in cases with superior breaks was significantly lower in group B (0%) compared to group A (25%, *p* = 0.048, Fisher exact test).

**Table 3:** Comparison of persistent SRF at postoperative 24 weeks in patients with different location of breaks.

	Location of breaks	Subjects	Persistent SRF	*p* Value
Group A	Superior break	12	3	0.292^a^
	Inferior break	10	4	
	Both	6	4	
Group B	Superior break	13	0	0.012*^a^
	Inferior break	5	1	
	Both	5	3	

^a^Fisher exact test.

### The side effects of air injection during SB surgery

In order to investigate the possible side effects of air injection, we had collected the clinical features and postoperative complications during the postoperative follow-up. As is showed in Table 4, at postoperative 24 weeks, all the RD in both group A and group B were reattached as suggested by indirect ophthalmoscopy, direct ophthalmoscopy and fundus photography. The postoperative BCVA increased in both groups. Although statistical difference was found in preoperative BCVA and preoperative IOP between group A and group B, no difference was found in terms of postoperative BCVA (*p* = 0.33) and postoperative IOP (*p* = 0.57). The common postoperative complications were recorded at every follow-up visits. In terms of postoperative macular epiretinal membrane (ERM), complicated cataract, new retinal tear, bleeding and choroidal detachment, no event was observed in both groups. However, the events of elevated IOP were found during the follow-up period, and treatment had been taken immediately if the elevated postoperative IOP was monitored.

**Table 4:** Postoperative clinical characteristics and complications.

	Group A (n = 28)	Group B (n = 23)	*p* Value
**At postoperative 24 weeks**			
Primary reattachment (%)	100%	100%	
BCVA (Log MAR)	0.35 ± 0.17	0.41 ± 0.14	0.33^a^
IOP (mmHg)	15.76 ± 2.04	15.20 ± 2.26	0.57^a^
**Complications during follow-up**			
The events of elevated IOP			
> 21 mmHg	5	4	
> 30 mmHg	1	1	
> 40 mmHg	0	0	
The epiretinal membrane	0	0	
Complicated cataract	0	0	
New retinal tear	0	0	
Bleeding	0	0	
Choroidal detachment	0	0	

^a^*t*-test.

BCVA, Best Corrected Visual Acuity; IOP, Intraocular Pressure.

### The potential risk factors for persistent SRF

The potential risk factors for persistent SRF at postoperative 24 weeks were investigated. Demographic characterizes (gender and age), co-existing diseases (hypertension and diabetes) and preoperative clinical features (disease chronicity, BCVA, IOP and PVR grading) were analyzed with binary logistic regression. As were presented in Table 5, no risk factor was found. These factors had no statistical relationship with the persistent SRF.

**Table 5:** Binary logistic regression of risk factors for persistent SRF at postoperative 24 weeks.

Factors	OR (95%CI)	*p *Value
Gender	14.96 (0.28–589.46)	0.149
Age*	0 (0–0)	0.309
Disease chronicity^#^	0.088 (0.001–12.814)	0.633
Diabetes	0.019 (0–17.88)	0.258
Hypertension	0.266 (0.006–11.593)	0.266
Preoperative BCVA	0.002 (0–4.503)	0.114
Preoperative IOP-	1.2 (0.813–1.771)	0.358
The PVR grading	0.151 (0.001–16.78)	0.432

Age*: Level 1, below 30 yrs; Level 2, above 30 yrs and below 60 yrs; Level 3, above 60 yrs.

Disease chronicity^#^: Acute: symptom duration <  = 7 days; Subacute: symptom duration > 7 days and <  = 30 days; Chronic: symptom duration > 30 days.

OR, odd ration; CI, confidence interval.

## Discussion

This present study was a retrospective preliminary study to investigate the effect of air injection in macular-involving RD during the SB surgery. In the study, as was mentioned above, group B had significantly worse BCVA, lower IOP and severer PVR grade before surgery, the disease severity in group A seem to be milder than group B, the incidence of postoperative SRF was supposed to be lower in group A. In opposite, our results showed that SRF in group A resolved more slowly, and the incidence was higher. There is a high probability that air injection during SB is benefit for SRF absorption. Moreover, the incidence of persistent SRF was the lowest in cases with superior breaks compared with the cases with inferior breaks or both breaks, and the incidence in cases with superior breaks was also significantly lower in group B compared to group A. The results might indicate that air injection helped the absorption of SRF in eyes with superior breaks. In addition, air injection during SB did not increase the event of ocular complications. Lastly, we performed logistic regression to identify risk factors for persistent SRF at postoperative 24 weeks, and no risk factor was identified. These findings indicated that air injection during SB was benefit for SRF absorption, and are particularly encouraging to the surgeons in that the air bubble may be effective and safe in helping fluid absorption for macular-off RD, especially in the cases with superior break.

Multiple factors may contribute to the presence and absorption of SRF after SB surgery. Previous studies reported that the prevalence of persistent SRF varied from 0 to 94% following successful surgery in RD patients^8–10^. First, the type of surgery performed was considered to relate to the incidence of persistent SRF, which was reported to be 53–94% following SB surgery and 0–40% following vitrectomy with the first OCT^11–16^. Second, the use of cryotherapy had been reported to be associated with the SRF occurrence due to the breakdown of blood barrier^17^. Third, incomplete drainage is another factor to be considered^3^. Until now, there was no study to investigate the effect of air bubble on SRF reabsorbing.

In our study, we grouped patients according to the use of air injection. The incidence of SRF in our study was 75% with the first postoperative OCT (at 1 week after SB without air injection), which was within the range of the previous studies, however, it decreased to 34.8% in the patients who had been undergone air injection. At postoperative 24 weeks, although all the retinas seem to be reattached on direct ophthalmoscopy by slit lamp observation in both groups, the Kaplan–Meier graph showed that incidence of persistent SRF in group A was significantly higher than that in group B. Moreover, among the group B, the patients with superior breaks had the lowest incidence (0%) than other patients with inferior or both breaks. As was presented in results, confounding factors such as age, sex, retinal tears, extend of buckle and viscosity of SRF were matched between group A and group B. The effect of cofounding factors like encircling and drainage could also be excluded, because all patients were treated with encircling and drainage by the same surgeon. Collectively, the results showed that air injection might significantly accelerate SRF absorption, and reduce the incidence of postoperative SRF after SB surgery, especially for patients with superior breaks. In further, except for IOP elevation, we did not observe other postoperative complication related with air injection, such as macular epiretinal membrane, complicated cataract, new retinal tears, and bleeding, which indicated air injection might be a safe procedure if performed meticulously.

The mechanisms of persistent SRF following RD surgery are still poorly understood. Oncotic pressure, hydrostatic pressure, and retinal pigment epithelial (RPE) pump are reported to be related with fluid absorption^18^. Several clinical researchers believed that although the subretinal fluid would gradually absorb when the retinal tears are closed, the SRF composition such as fibrin and cell debris were concentrated, resulting in increased fluid cellularity and viscosity^19,20^. Especially for the longstanding detachment, the high viscosity of the SRF might stick to the RPE, the clogging of the RPE had been demonstrated to be a contributing factor for persistent SRF^21^. In addition, the procedures such as cryotherapy, episcleral buckle, and drainage during SB surgery may affect the ocular blood circulation^17,22^, causing the decrease function in retinal and choroidal capillaries, obstruction of vortex venous return, damage to the blood-retinal barrier, resulting in RPE malfunction^23–25^. And such changes may be contributed to the slowed absorption of SRF. In our study, it was unclear that how air bubble contributed to the SRF absorption. The possible causes might be as following; firstly, the surface tension of the air bubble had pressure on the fovea and allowed the neuroepithelial layer to adhere to the pigment epithelium. Secondly, the raised IOP during and after air injection may help the SRF to be drained through the sclerotomy site more meticulously than in cases without air injection. Lastly, circumferential encircling procedures might result in redundancy of retina, and the redundancy that developed at the break (also was referred to as fish mouthing), allowed a pathway for fluid to continue leaking from the vitreous cavity into the subretinal space. And intraocular air bubble might be a good strategy to iron out the fish mouth conveniently. Therefore, air injection should be considered when performing circumferential encircling in macular-off RD.

It is controversial whether persistent SRF would damage the final visual acuity. Some authors reported that SRF may not affect the final postoperative vision and anatomical retinal reattachment^8,14,16^. However, most authors agreed that persistent SRF did delay VA recovery^2,3,26^. Especially for the central vision, loss of depth perception and metamorphopsia may persevere for a long time even after successful SB surgery. Furthermore, similar to central serous chorioretinopathy (CSC), progressive photoreceptor damage and irreversible vision loss might occur due to persistent SRF^27,28^.In the present study, postoperative BCVA increased significantly in both groups compared with pre-operative BCVA, however, there was no statistical difference between the two groups. And we did not investigate the relationship between persistent SRF and the final VA. Nevertheless, the possible adverse effect of persistent SRF could not be ignored.

However, several limitations need to be considered in the present study. First, the small sample size and retrospective observational study design are the drawbacks, Second, although all the SB surgery were done by one surgeon, the procedure was still varied. For example, the volume of drainage, the localization of cryotherapy, conjunctival incision, buckling materials, and buckling configuration were personalized based on the clinical features of RD, which gives rise to the potential confounders. The use of air injection was an accessory technique and was determined by the intraocular pressure during the surgery, which may lead to selection bias when grouping the patients. Lastly, the present study only investigated the subretinal fluid at the macular area, while the fluid at the peripheral retina was not included. OCT scanning with wider area should be performed in the further study. Herein, in order to further determine the effect of air injection on SRF absorption, a randomized controlled trial with a large size subject is further required in the future study.

In summary, the study indicated that air bubble might facilitate SRF absorption, especially for the longstanding macular-off RD with superior breaks, these cases are most likely to benefit from SB with air injection. Air injection as an accessory technique should be recommended when performing encircling during SB surgery.

## Methods

We retrospectively reviewed the medical record of patients with primary rhegmatogenous macular-involving RD at Guangxi Ophthalmic Center between January 2018 to December 2019 and enrolled 51 patients who had been undergone SB surgery. These subjects were categorized into two groups based on the use of air injection, group A consisted of 28 cases who had been undergone SB without air injection, group B consisted of 23 cases who had been undergone SB with air injection. Inclusion criteria were: (1) treatment naïve when presented to our hospital; (2) the macular was involved; (3) at least 6 months follow-up period after operation. Exclusion criteria were as the follows: (1) history of ocular surgery; (2) history of ocular trauma; (3) macular diseases, such as macular hole, macular epiretinal membrane, macular oedema, and age-related macular degeneration. The study was performed in accordance with the Declaration of Helsinki. Written informed consent was required from the participants, the dataset of clinical feature and images of ophthalmic examination will be collected in the study, and identifiable human material or patient data will not be included to maintain confidentiality. Ethics approval for this retrospective, comparative review (No. KY-GZR-2019–053) was obtained from the People’s Hospital of Guangxi Autonomous Region, a teaching hospital with ethical committee. The study was conducted in accordance with the Declaration of Helsinki.

The patients had been received comprehensive ophthalmic examinations at preoperative and postoperative visits, including best-corrected visual acuity (BCVA), intraocular pressure (IOP), slit-lamp, indirect and direct ophthalmoscopy, and OCT scan (Topcon, 3D OCT-2000, Tokyo, Japan). The clinical features (the clocks of RD, retinal tears, RD extent, and Proliferative vitreoretinopathy (PVR)) were evaluated and determined according to the fundus images and medical records by the same consultant. All subjects performed by the same experienced surgeon (Dr HB Zhong) were selected. Buckling materials and localization were placed accurately and adequately in all surgeries. All patients were treated with encircling to relieve the forces of vitreous traction. A segmental silicone tire was placed and sewn to keep it in place. The effective buckle was extended sufficiently around the retinal break. The buckle should extend 3 mm posterior to the break and 1.5–2 mm laterally on either side. Then the eye wall was indented with the minimal buckle height, which is necessary to achieve closure of the break. Cryotherapy and external subretinal fluid drainage were also carried out in all patients. Drainage puncture was performed with 23-gauge needle at an area with relatively deep subretinal fluid away from choroidal vessels, the site of drainage was selected using indirect ophthalmoscopy and always beneath the silicone tires. And the indirect ophthalmoscopic evaluation assessed that the subretinal fluid was almost completely drained. The drainage of SRF was considered to be effective when it placed the retinal breaks in juxtaposition to the buckle. Moreover, the surgical sequence was cryotherapy, SRF drainage and other surgical manipulation of the globe, air injection was performed at the end of the operation, the amount of injected air ranged from 0.5 to 0.8 ml. In addition, they had kept face-down position for at least 12 h following surgery.

The presence of SRF and postoperative ERM were recognized and determined by OCT examination, which was performed with 5LineCross and 3D scan mode. Scan length and width were 9.0 mm × 9.0 mm with horizontal and vertical direction. SRF was defined as exudate or fluid between the neurosensory retina and the RPE in the fovea. Postoperative ERM was defined as hyper-reflective line above the inner surface of macular, and ERM at stage 3 or stage 4 was included in the present study. The demographic characteristics were collected, including age, gender, and symptom duration. The clinical features at the first presentation and every postoperative follow-up visit was abstracted for use in this study. Statistical analysis was performed using SPSS (version 20.0, Chicago, IL, USA). The values of continuous variables (age, symptom duration, BCVA, and IOP) were presented as mean ± standard deviation (SD). After been tested for normal distribution, the BCVA, IOP and age were compared with independent t-test, while symptom duration was compared with Mann–Whitney U-test. Categorical variables were presented as the number of subjects and its percentage, and the difference was analyzed by Chi-square test or Fisher exact test. A Kaplan–Meier graph comparing survival time of SRF for the air injection group and no air injection group was plotted. Binary Logistic Regression was used to investigate the associated factors for the persistent SRF. *p *Value < 0.05 was considered statistically significant.
